# Can Guided Discovery Instruction Be Detrimental to Children with Different Levels of Aquatic Competence in Infancy?

**DOI:** 10.3390/children11070854

**Published:** 2024-07-14

**Authors:** Juan Antonio Moreno-Murcia, Luciane de Paula-Borges, Alfonso Trinidad

**Affiliations:** 1 Departamento de Ciencias del Deporte, Universidad Miguel Hernández de Elche (España), Avinguda de la Universitat d’Elx, s/n, 03202 Elche, Alicante, Spain; 2 Centro de Enseñanza Samaniego (España), Calle Pago de las Viñas, s/n, 30820 Alcantarilla, Murcia, Spain; luciane@cesamaniego.com; 3 Departamento de Educación e Innovación Educativa, Aqualab Research Group, Universidad Europea de Madrid (España), C. Tajo, s/n, 28670 Villaviciosa de Odón, Madrid, Spain; alfonso.trinidad@universidadeuropea.es

**Keywords:** teaching, methodology, learning, school children, teacher, aquatic competence

## Abstract

Repetitive practice can become an exploratory activity where instruction and discovery are linked, allowing instruction and guidance through tasks that help to construct and acquire the knowledge and skills that make up the content. (1) Background: The aim of the study was to show how a teaching method based on guided discovery would affect the teaching of children’s aquatic competence in schoolchildren with different levels of competence. (2) Methods: An observational study was conducted with 385 schoolchildren (195 boys and 189 girls) aged 3–5 years belonging to a charter kindergarten, using an Instrument for the Measurement of Aquatic Competence in Children (SMACC) consisting of 17 items grouped into three dimensions: socio-affective, cognitive, and motor. (3) Results: After measuring aquatic competence, all age groups and all variables (motor, cognitive, and socio-affective) showed differences between pre- and post-scores and a high magnitude of effect size. When the teaching intervention was analyzed according to the level of aquatic competence of the age group, improvements were found in all variables in both the low and high-competence groups. (4) Conclusions: This study describes how guided discovery instruction has equal effects at different levels of proficiency. Furthermore, when this type of instruction was used, aquatic competence was explained not only by the motor and socio-affective dimensions but also by the cognitive variable.

## 1. Introduction

Discovery learning without teaching assistance can be less efficient for students, especially those with prior knowledge deficiencies or learning difficulties. Kirschner et al. [1] argue, based on cognitive load theory, that understanding a problem imposes a significant burden on working memory, limiting a student’s ability to engage in complex reasoning due to the scarcity of cognitive resources. Therefore, the direct instruction model, which does not provide a structured approach to addressing working memory limitations, may fall short of achieving certain educational goals. Conversely, this model offers a progressive guide for students, leading them from broader tasks to more analytical ones, and providing necessary step-by-step assistance.

Discovery learning also incorporates a solid foundation through direct instruction, enabling students to acquire basic skills and develop complex competencies. According to Trninic [2], it facilitates awareness of underlying principles, while Montanero et al. [3] note its effectiveness in fostering conceptual change. However, there is a lack of scientific evidence confirming the impact of guided discovery on students with varying initial competency levels. This raises the question: does guided discovery instruction have the same effects on students with different levels of prior knowledge?

Additionally, certain factors can influence retention, feedback, practice objectives, and session structure [4]. Ideally, practice should be continuous, without significant breaks, to ensure skill retention. In non-linear pedagogy, didactic games for understanding, which manipulate key task constraints, can facilitate functional movement patterns and decision-making behaviors, guiding learning progression in Physical Education [5].

The debate on the most effective teaching methodologies has often been inconclusive. Various methods are combined based on learning objectives and context, leading to two main instructional methods: direct instruction and discovery learning. Direct instruction is characterized by structured teaching, providing conceptual and procedural information with high teacher control over activities [1,6]. This method includes frequent scaffolding that gradually decreases, enhancing learner autonomy [7]. Procedural steps are explained and practiced, and feedback is given as needed [8]. Tasks are systematically guided through individual and group work.

In contrast, discovery learning, as described by Bruner [9], involves students investigating independently, seeking information, exploring, and discovering ideas through various resources. This method is not ideal for reproducing knowledge [10]. The degree of assistance varies with the student’s maturity and the discovery type [2]. According to Mosston and Ashworth [10], it involves a logical sequence of questions leading to the discovery of predetermined concepts or principles.

### 1.1. Effectiveness of Teaching Styles

Teaching methods should make learning meaningful by connecting new information with students’ prior knowledge. If not, students may resort to rote memorization without understanding [11]. Effective knowledge retention requires students to actively think about their learning, necessitating significant interactivity between teacher and student.

Direct instruction is often deemed more effective from a cognitive perspective than discovery learning [1,11]. However, Herman and Gomez [12] suggest that direct instruction may be less motivating, potentially reducing its effectiveness. Therefore, the motivational aspect and social context should be considered.

Montanero et al. [3] found that discovery learning could yield better results in depth and functionality of knowledge rather than quantity. For novice learners, direct instruction is more effective, but it can be counterproductive for experienced learners, as Sweller et al. [13] describe in the “expertise reversal effect”, where methods effective for novices may hinder experienced learners.

As students advance, the teacher’s role should become more secondary, intervening only when necessary. Initially, facing a problem requires prior explanation and examples for support. Alfieri et al. [14] argue that students with minimal knowledge might solve new problems independently before receiving explanations, suggesting the limited effects of unsupported discovery tasks.

An optimal teaching approach should include:

(a) Structured guided tasks (scaffolding) to assist students [15],
(b) Tasks requiring students to explain their ideas accurately with timely feedback, or
(c) Tasks offering practical examples for success.

### 1.2. The Present Study

Trninic [3] suggests that repetitive practice can become an exploratory activity, integrating direct instruction and discovery. In such practice, the teacher guides tasks, making learners aware of knowledge and skill construction. This teaching approach has proven effective in facilitating knowledge and skill acquisition. The Comprehensive Aquatic Method [16] has shown optimal results in aquatic education [17].

The present study aimed to investigate the effects of guided discovery instruction on the competence levels of 3-, 4-, and 5-year-old students. Using the Comprehensive Aquatic Method, we assessed its impact on children’s aquatic competence in a school setting. We hypothesized that:

Guided discovery instruction would result in learning regardless of the student’s initial competence level.

The level of aquatic competence achieved would be primarily explained by the motor domain.

## 2. Materials and Methods

### 2.1. Participants

The sample consisted of 385 students (195 boys and 189 girls) aged 3 to 5 years (M = 4.32 years; SD = 1.09), belonging to a Spanish subsidized early childhood education center. A total of 217 students were 3 years old, 123 were 4 years old and 135 were 5 years old. Regarding aquatic experience, the 3-year-old group started the program with no experience, the 4-year-olds with one year of experience in a weekly class in the previous school year, and the 5-year-olds with two years of experience in a weekly class during the school year. The present investigation has the approval of the Human Research Committee of the Miguel Hernández University of Elche (Spain) (2019.286.E.OEP). Informed consent for the study was signed by all parents of the children.

### 2.2. Materials and Instruments

To measure aquatic competence, we used the Instrument for Measuring Aquatic Competence in Children (SMACC) by Moreno-Murcia et al. [18]. It consists of seventeen items grouped into three dimensions: socio-affective, composed of five items; cognitive, composed of five items; and the motor area, composed of seven items. A rubric was used as a five-item rating system for the infant’s behaviors. For example, in the item that has to do with breathing (when asking children in a shallow area to blow bubbles underwater by expelling air through the mouth and nose), 1 corresponds to “Blow without touching the water with the face”; 2 corresponds to “Blowing only with the mouth at the water level”; 3 to “Not blowing into the water, but putting the face completely into the water”; 4 to “Blowing through the mouth and nose, putting the face completely into the water”; and 5 to “Is able to coordinate breathing (taking in and expelling air) with movements continuously several times”. In the cognitive area, when participants are instructed to play tag, the teacher will indicate which part of the body they have to touch on others to consider them tagged and with which part of their body they have to touch. The number 1 corresponds to “Is not able to perform any action”; 2 corresponds to “gets confused with the names of body parts”; 3 to “can only identify parts of their own body”; 4 to “can identify parts of their own body and those of their peers”; and 5 to “demonstrates confidence and initiative in performing the activity and shows knowledge of body parts, both their own and those of their peers”. In the socio-affective area, when measuring self-control, when the child is asked to enter the water by jumping in, The number 1 corresponds to “refuses to enter the water by jumping”; 2 to “expresses refusal by crying”; 3 to “performs the activity but with the help of the teacher or an adult”; 4 to “is able to jump into the water without the teacher’s help”; and 5 to “is able to jump from various heights and in different ways”. The internal consistency of each dimension was 0.94, 0.91, 0.89 in take 1, while in take 2 it was 0.95, 0.87, and 0.79. A global dimension of global aquatic competence formed by the three dimensions was also contemplated, which obtained a Cronbach’s alpha of 0.97 for take 1 and 0.95 for take 2. The children were assessed by trained evaluators from the research team.

### 2.3. Procedure

The intervention focused on achieving aquatic competence, which includes the development of basic aquatic skills. These skills are designed to help children gain confidence and autonomy in the aquatic environment, as well as the ability to perform a variety of basic movements in the water.

The Comprehensive Aquatic Method was applied, where the experimenter uses an active teaching approach through discovery learning with a clear support structure for the learner, occasionally resorting to direct instruction depending on the situation [18]. A typical aquatic activities class consisted of an assembly at the beginning to discuss what was done in the last session, which allowed for a connection between previously applied content and what would be addressed in the current session, seeking a relationship between contents. Then, in the second part of the class, the contents were experienced through proposed activities via games. The class ended with a low-rhythm game or an assembly, depending on the theme proposed in the class, or a necessary reflection on certain attitudes and/or a proposal for the next class. The duration of each part of the class depended on the student’s enjoyment or the need to change the game or activity.

During the intervention, since the Comprehensive Aquatic Method involves active engagement of the student, a series of motivational strategies were applied to support student autonomy. These included explaining the purpose of the activities at the beginning of the class, recognizing individual and collective achievements, fostering relationships among participants and their autonomy in choosing games and making decisions, using flexible and clear language that acknowledges and identifies with the children’s emotions, and seeking intrinsic motivation through activities, among other strategies. The idea was for students to gain prominence over time and learn to manage their own enjoyment and learning autonomously.

One week prior to the start of the intervention, measurements were taken on the participants. They were informed that they would be part of a study “seeking a new way of teaching in the aquatic environment”, but were not told the exact details of the study proposal. At the end of the four-month intervention, they were measured again. As for the content of the training units, they were oriented towards the acquisition of fundamental aquatic skills.

### 2.4. Data Analysis

Cronbach’s alpha coefficient was used to verify the internal consistency of each factor. To answer the research questions, a repeated measures ANOVA (Level of aquatic competence × Time) was conducted with all dependent variables (motor, cognitive, and socio-affective). Data analysis was performed with the SPSS 22.0 statistical program.

## 3. Results

To test the effect of the active methodology, a repeated measures analysis was carried out by age groups and the variables motor, cognitive, socio-affective, and aquatic competence. In all ages and in all variables (Table 1) there were improvements (*p* < 0.001) from pre to post intake, with values of a high magnitude (<0.8) in the effect size obtained.

To carry out the analysis of the results of the study in terms of the level of competence by age distributed in two groups (low competence and high competence), these were calculated taking into account the median obtained at each age. In this way, the participants were classified into two groups by age, with a cut-off difference of 1.42 at age 3, 3.2 at age 4, and 3.72 at age 5. As can be seen in Table 2, after the repeated measures analysis with the variables by age groups and level of competence, in all ages (3, 4, and 5 years) there were improvements (*p* < 0.001) from the pre- to post-acquisition in all variables (motor, cognitive, and socio-affective), both in the low competence group and in the high competence group. Thus, there is no determinism in low proficiency learners to be able to benefit from active methodologies, since the starting state is not determinant for the reported possible benefit.

With the intention of testing, both with the data from take 1 and the data from take 2, which variables had the greatest impact on the dependent variable (aquatic competence) of a set of independent explanatory variables (motor, cognitive, and socio-affective), a linear regression analysis was carried out (Table 3). After the regression analysis with the pre-data, aquatic competence was explained by 90% of the variance, specifically by the motor and socio-affective variables, while with the post-data, the variance explained increased to 99%, with all the independent variables being predictors, with the motor and socio-affective variables having a greater weight.

## 4. Discussion

The aim of the present study was to show the effect of guided discovery instruction through an intervention in the teaching of aquatic competence to children aged 3, 4, and 5 years with different levels of competence. It was confirmed that, regardless of the child’s level of competence, guided discovery instruction produced learning in the two groups analyzed. However, the idea that the amount of aquatic competence achieved after instruction with this type of teaching was mainly explained by the motor area was rejected.

The scientific literature has pointed out that there are two types of instructional methods that have been combined with learning objectives and context, giving rise to direct instruction [1,6] and discovery learning [9], the latter carried out with and without teacher support. However, there are differences in the literature on how discovery learning has shown better results than direct instruction [3], without it being clear which of the two is more effective. For example, Alfieri et al. [14] and Kirschner et al. [1] suggest that direct instruction is more effective from a cognitive point of view than pure discovery learning. Moreover, in a meta-analysis of 164 studies, Alfieri et al. [14] found that most discovery learning experiences generated better learning outcomes than other unstructured teaching strategies but worse outcomes than most direct instruction sequences. As Montanero [19] points out, this supposed disadvantage compared to direct instruction may be due, on the one hand, to the fact that a generally loosely guided approach generates an excessive cognitive load that makes it difficult to learn complex knowledge and skills [1]. On the other hand, discovery learning is not well suited to the learning style of many students [20].

Conversely, some authors argue that unsupported inquiry learning would be less efficient in students with low proficiency, as indicated by cognitive load theory [1]. The study data showed that after comparing students with different levels of competence (low and high), no differences were found in learning aquatic competence between the ages of 3 and 5 years. This contradicts the claim about how prior knowledge is a determinant of successful learning, whether in students who are learning for the first time or who already have prior knowledge [14]. Moreover, the data from the present research are also consistent with other claims about how direct instruction or teacher-delivered support within discovery or inquiry can lead to student acquisition of both basic skills and the development of more complex competencies [2,3]. However, no further scientific evidence has been found within the literature to corroborate the effect of this type of practice according to the initial competency level of the learner.

Another important question about teaching methods is how students learn in order to assimilate and understand the tasks. In this line, scientific literature has corroborated how the student should avoid memorization without comprehension [11]. In relation to the study, it was demonstrated how the student’s cognitive involvement increased during the learning of aquatic competence through the guided discovery method after the intervention. This could be related to the interaction of the teacher with the student, the teacher’s concern for the student to think about what he/she learns, and making the student motivated to learn. These factors could help in knowledge retention, thus coinciding with the statements made by Duschl and Duncan [21] on how the discovery method could be more effective as long as it is guided and directed by the teacher.

On the other hand, the literature highlights the role of motivation and the use of instructional models in student learning. For authors such as Herman and Gómez [12], the use of these methods makes the student less motivated and has less effect on the development of tasks. In this study, it was observed that through the guided discovery method, aquatic competence improved mainly in the involvement of students at the socio-affective level rather than at the motor level. This contradicts the assertion that instruction can reduce learner motivation. Moreover, this motivational dimension should be taken into account as a conditioning factor in student learning within aquatic competence in view of the present intervention.

## 5. Conclusions

This research helps Physical Education professionals or aquatic educators to see that regardless of the student’s level of competence, the acquisition of aquatic skills can be carried out using the discovery model guided by the teacher. In this sense, the tasks could be broken down into parts or presented in different parts, so that as the aquatic competence progresses, feedback can be given to motivate the student. However, it would be interesting in the future to carry out studies with a larger sample and include a control group. This would rule out other possible causes explaining the improvement observed through partial guided discovery teaching of the teacher using the Comprehensive Aquatic Method, and ensure the results were not influenced by other uncontrolled events and maturation, the practice effect, or regression to the mean.

This study describes how teacher-guided discovery instruction in the teaching of children’s aquatic competence has the same effects on students starting from different levels of knowledge. Likewise, aquatic competence improves student involvement mainly at the socio-affective level rather than at the motor level and may therefore be a conditioning factor in student learning within aquatic competence.

## 6. Limitations

Potential limitations of this study include generalizability, as the research was conducted in a specific center with a specific demographic group (children aged 3–5 years), which may limit the applicability of the findings to other settings or age groups, especially older children or in different educational settings. The reliability of the SMACC, the measurement instrument used to assess aquatic competence in motor, cognitive, and socio-affective dimensions, may vary between different settings or populations, which could affect the robustness of the study results. Observer bias in observational studies may also influence data collection and interpretation, potentially confounding results and suggesting the need to discuss measures to minimize this bias. Potential confounding variables, such as individual differences in learning styles, previous experience with aquatic activities, or external influences, may have affected the results and should be controlled for or discussed to strengthen the study. The duration and sustainability of the effects of the study focused on the immediate post-intervention effects on aquatic competence, without assessing the long-term retention of skills or the sustainability of these effects over time, aspects which, if discussed, would provide a more holistic view of the intervention. Addressing these limitations would provide a balanced perspective on the study findings and their implications for future research or educational practice related to children’s aquatic competence.

## Figures and Tables

**Table 1:** Repeated measures analysis with variable by age group.

		3 Years (*n* = 127)			4 Years (*n* = 123)			5 Years (*n* = 135)		
Variables		*M*	*SD*	η^2^	*M*	*SD*	η^2^	*M*	*SD*	η^2^
Motor skills	Pre	1.27	0.18	0.96	2.99	0.22	0.81	3.52	0.45	0.88
	Post	2.24 **	0.06		3.49 **	0.18		4.76 **	0.32	
Cognitive	Pre	1.40	0.34	0.99	3.34	0.39	0.86	3.43	0.26	0.96
	Post	3.60 **	0.08		4.37 **	0.08		4.95 **	0.15	
Socio-affective	Pre	1.75	0.28	0.98	3.22	0.51	0.90	4.03	0.26	0.94
	Post	4.24 **	0.28		4.72 **	0.21		4.97 **	0.08	
Aquatic competence	Pre	1.47	0.14	0.99	3.18	0.25	0.95	3.66	0.24	0.96
	Post	3.36 **	0.10		4.19 **	0.15		4.89 **	0.18	

Note: ** *p* < 0.001.

**Table 2:** Repeated measures analysis with the variables by age group and proficiency level.

		3 Years (*n* = 127)						4 Years (*n* = 123)						5 Years (*n* = 135)					
		Low Competence (*n* = 61)			High Competence (*n* = 66)			Low Competence (*n* = 61)			High Competence (*n* = 62)			Low Competence (*n* = 64)			High Competence (*n* = 71)		
Variables		*M*	*SD*	η^2^	*M*	*SD*	η^2^	*M*	*SD*	η^2^	*M*	*SD*	η^2^	*M*	*SD*	η^2^	*M*	*SD*	η^2^
Motorskills	Pre	1.13	0.05	0.99	1.39	0.17	0.96	2.91	0.21	0.99	3.06	0.20	0.85	3.14	0.34	0.90	3.87	0.19	0.96
	Post	2.22 **	0.07		2.26 **	0.05		3.43 **	0.24		3.55 **	0.05		4.65 **	0.44		4.85 **	0.02	
Cognitive	Pre	1.31	0.11	0.99	1.39	0.95	0.99	3.14	0.40	0.99	0.354	0.26	0.91	3.29	0.25	0.95	3.56	0.19	0.98
	Post	3.42 **	0.18		3.47 **	0.13		4.35 **	0.11		4.39 **	0.02		4.90 **	0.22		5.00 **	0.00	
Socio-affective	Pre	1.56	0.08	0.99	1.93	0.29	0.98	2.89	0.48	0.99	3.54	0.29	0.95	3.94	0.33	0.91	4.10	0.11	0.98
	Post	4.16 **	0.32		4.30 **	0.22		4.65 **	0.28		4.78 **	0.07		4.95 **	0.11		5.00 **	0.00	

Note: ** *p* < 0.001.

**Table 3:** Regression analysis of aquatic competence by areas (*n* = 385).

Variable	Predictor	B	SEB	b	∆*R*^2^
Take 1		2.39	0.03		0.90 **
	Motor skills	0.27	0.02	0.42 **	
	Cognitive	0.03	0.00	0.02	
	Socio-affective	0.35	0.02	0.54 **	
Take 2		0.56	0.01		0.99 **
	Motor skills	0.44	0.00	0.72 **	
	Cognitive	0.33	0.00	0.19 **	
	Socio-affective	0.45	0.00	0.65 **	

Note: ** *p* < 0.001.

## Data Availability

Data are contained within the article.
